# Aromatase Inhibitors to Augment Height: Continued Caution and Study Required

**DOI:** 10.4274/jcrpe.v1i6.256

**Published:** 2010-12-08

**Authors:** Mitchell E. Geffner

**Affiliations:** 1 Saban Research Institute, Childrens Hospital Los Angeles, Keck School of Medicine, University of Southern California, Los Angeles, USA; +001 323 361 70 32 +001 323 361 13 50mgeffner@chla.usc.eduChildrens Hospital Los Angeles, 4650 Sunset Blvd. Mailstop 61 Los Angeles, CA 90027

**Keywords:** Aromatase, inhibitors, gonadotropin-releasing hormone agonists, estrogen, androgen

## Abstract

Aromatase inhibitors (AIs) are a class of drugs that prevent conversion of androgens to estrogens, and that are approved in the United States as adjunctive treatment of estrogen receptor-positive breast cancer. Because ultimate fusion of the growth plates is estrogen-dependent in both boys and girls, AI administration may help to slow down epiphysial maturation and allow for greater height potential. Research trials in children with short stature have predominantly been done in Finland and Florida. Despite the apparent efficacy described by these groups, only ˜110 children worldwide have been treated with AIs in research protocols (and usually concomitant with other growth-promoting agents) as of the end of 2008 (and none to final height). That said, many children are being treated with AI’s in the United States outside of research protocols. Furthermore, little is known about the short- and long-term safety of AIs in children. Thus, it is imperative that there be well-designed, long-term studies of efficacy and safety of AI use in pediatric populations.

**Conflict of interest:**None declared.

## INTRODUCTION

With regard to physicians’ abilities to treat short stature, it has been just over 50 years since the first child in the United States (US) with growth hormone (GH) deficiency was treated with GH, initially of cadaveric pituitary origin (1). Since 1985, only recombinant human GH (rhGH) has been used to treat children with growth disorders of which there are now nine FDA-approved indications (some associated with GH deficiency and others with presumed GH resistance), the most controversial of which is idiopathic short stature (ISS). As highlighted in the recent book, Normal At Any Cost by Cohen and Cosgrove (2), there has been a push to create a taller society among parents and physicians. To accomplish such a goal, there is now available an expanding pharmacological repertoire that includes direct growth-promoting agents such as rhGH and, now, insulin-like growth factor-I (IGF-I) in the US and in Europe, and, historically, anabolic steroids, mostly used outside the US. An alternative approach to height augmentation employs agents that impede puberty and, in particular, estrogen production (in both sexes), which is responsible for ultimate epiphysial fusion. This approach has, traditionally, employed gonadotropin-releasing hormone (GnRH) agonists (GnRHa) and, more recently, aromatase inhibitors (AIs). These approaches have been used as sole treatments or in various combinations, with varying efficacy and safety profiles. 

For example, in a study by Yanovski et al from US, use of a GnRHa alone in 26 short adolescent males with normally timed puberty for a mean of 3.5 years increased height by 0.6 SD, but substantially decreased bone mineral density (BMD) (3). Carel from France in 2006 wrote that, although long-term use of a GnRHa alone, when used outside the context of precocious puberty, yields some height gains and that GH alone modestly increases adult height in short adolescents with ISS or in those born small for gestational age (SGA), combination therapy lacks proof of additional efficacy (4). In that vein, a Dutch study of combined GH and GnRHa therapy failed to show any difference in final height after 3 years (compared to a no-treatment control group) in either short adolescent males born SGA or with ISS (5). However, Tanaka from Japan recommends combined GH and GnRHa treatment in short GH- or non-GH-deficient children who start puberty at a short height (6). 

Thus, it seems that most investigators do not advocate routine combination growth-promoting therapy for normal short children, but espouse the need for further study via large randomized controlled trials to assess efficacy and safety, as well psychological benefits and economic viability. That said, with the availability of AI’s, the first new class of potential oral growth-promoting agents, many short (or predicted to be short) children are being treated with this class of drug, either as mono-therapy or as part of multi-drug regimens.

## PATIENT 1

To understand the rationale for AI treatment, it is important to first review the historical understanding of the general physiological roles of androgens and estrogens in the 1980s. In that era, it was thought that, in males, testosterone was the principal sex hormone responsible for the pubertal growth spurt, skeletal maturation, accrual of bone mineral, and maintenance of skeleton (anti-osteoporotic action). Conversely, it was believed that estrogen was not an important regulator of follicle stimulating hormone (FSH) secretion and had a trivial role in non-reproductive tissues. Finally, it was also believed that local conversion of testosterone to estradiol in the brain exerted an important effect on psychosexual differentiation (7). However, the identification of two men, one with a mutation of the estrogen receptor gene (8)and another with a mutation of the aromatase gene (9), taught us differently. Similarities and differences (in italics) between these two pristine cases are described in Table 1 (9). That both cases were associated with lack of estrogenic signaling caused a paradigm shift underscoring the critical role of estrogen (presumably in both sexes) in epiphysial maturation/closure and on gonadotropin regulation.

**Table 1:**
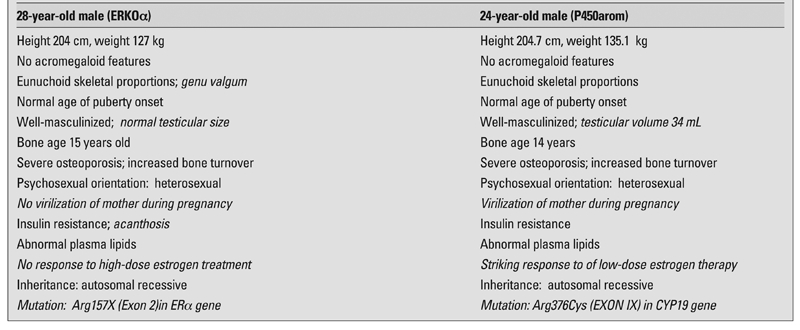
Comparison of estrogen receptor-α deficiency (ERKOα) and of aromatase deficiency

## AROMATASE PHYSIOLOGY

Furthermore, these cases brought to light the possibility of a new understanding of physiology which could be applied to growth manipulation in boys predicted to have short adult height, i.e., intentional pharmacological blockade of aromatase-driven conversion of androgens to estrogens. To better understand this rationale, it is critical to note that the human aromatase enzyme is a cytochrome P450 hemoprotein-containing enzyme located in the endoplasmic reticulum of estrogen-producing cells located in many tissues. These include osteoblasts and chondrocytes in bone, stromal cells of fat, Leydig and germ cells of testes (in males), smooth muscle cells of the vasculature, and several areas of the hypothalamus, limbic system, and cerebral cortex. Note that the aromatase enzyme catalyzes the rate-limiting step in conversion of androgens to estrogens and is encoded by a single gene, CYP19, located on chromosome 15q21.2 (10).

Conveniently, the class of drugs known as AIs was already on the market in the US to adjunctively treat estrogen receptor-positive breast cancer, their sole FDA-approved indication. More specifically, AIs prevent conversion of the C19 androgenic steroids, androstenedione (A) and testosterone (T), to their C18 estrogenic counterparts, estrone (E1) and estradiol (E2), respectively. They also block conversion of estrogens to catechol estrogen, 2-hydroxyestrogen, and 6a-hydroxyestrogen, metabolites which may have critical roles in induction or promotion of estrogen-responsive malignancies. There are two classes of AIs, non-steroidal and steroidal, as well as three generations. The former reversibly bind to the heme moiety of the cytochrome P450 aromatase enzyme, while the latter are derivatives of A that act irreversibly as false or “suicide” substrates for the aromatase enzyme (11). The categorization of AIs can be found in Table 2, along with clinical comments about their toxicity. The generational differences among the AIs show that, with newer generations, progressively lower doses are needed to achieve a higher efficiency of enzymatic blockade (Table 3).

**Table 2:**
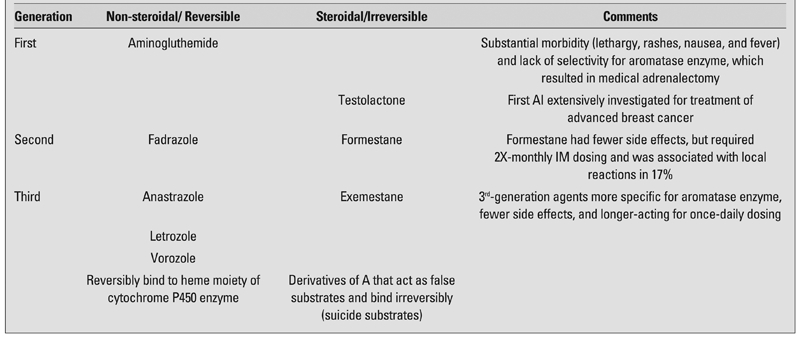
Aromatase inhibitor classes

**Table 3:**
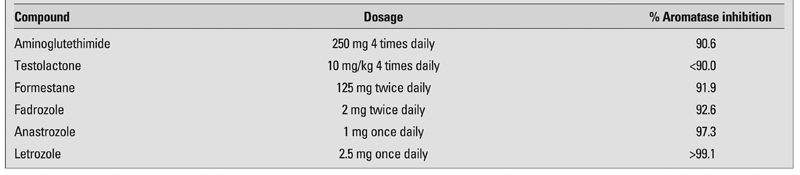
Relative Potency of select aromatase inhibitors

## USE OF AROMATASE INHIBITORS IN CHILDREN

Critical research in the pediatric literature as it applies to AI use for height purposes will be reviewed in temporal sequence. Other clinical conditions in childhood in which AIs have been used, e.g., McCune-Albright syndrome, testotoxicosis, congenital adrenal hyperplasia, and pubertal gynecomastia, will not be discussed (12, 13, 14, 15, 16). 

The first reported use of AIs to assess their effect on height (17) was in a group of 33 Finnish adolescent boys (mean age 15 yr) with constitutional delay of growth and puberty (CDGP) who were randomized to receive either: intramuscular depot T (1 mg/kg every 4 weeks for 6 months) and letrozole (2.5 mg/d) for 12 months (n=11); T and placebo for 12 months (n=12); or no treatment (n=10). After 12 months, serum E2 levels in those patients on letrozole were maximally suppressed and serum T levels were maximally increased. Mean bone age (BA) advancement was desirably and significantly less in the group treated with T and letrozole (only 0.9 years) vs T and placebo (1.7 years) and no treatment (1.1 years). This difference resulted in a mean increase in predicted adult height of 5.1 cm in the group receiving T and letrozole, which was significantly more than in the other two groups.

The first report of AI use in children from the US in 2003 described the pharmacokinetic properties of exemestane in 12 healthy eugonadal adolescent and young adult males between the ages of 14-26 years of age over a 10-day period (18). The investigators found that 25- and 50-mg doses of exemestane had similar effects on all parameters measured, including estrogen (32-38% decrease), testosterone (˜60% increase), sex hormone binding globulin (SHBG) (19-21% decrease), and dehydroepiandrosterone (DHEA) sulfate (no change). Further studies to investigate pharmacokinetics of a single oral 25-mg dose of exemestane noted peak absorption at 1 hour with a terminal half-life of 8.9 hours. In addition, mean serum E2 levels were maximally suppressed at 12 hours and returned to baseline after 3-6 days.                       

In a retrospective study of 18 non-testosterone-treated adolescent males with limited growth potential (mean age 15 years) and rapidly advancing BA, Karmazin et al reported that treatment solely with letrozole, 2.5 mg orally daily (mean duration 12.4 months), resulted in a slow down in BA advancement compared to the rate seen prior to initiation of treatment (mean change in BA to chronological age = 0.68 vs 1.57 years), thus explaining an increased mean predicted adult height in the letrozole-treated group (-0.64 vs -1.41 SD) (both p<0.0005) (19). 

In a follow-up study of the previously described group of Finnish boys with CDGP, Hero et al reported mean near-adult heights of 175.8 cm and 169.5 cm (p=0.04) and mean gains in height SD scores of 1.4 and 0.8 (p=0.03) in the T and letrozole vs the T and placebo groups, respectively (20).

In the largest and longest US study to date, Mauras et al randomized 52 adolescent males with GH deficiency to treatment with GH and either daily co-treatment with anastrozole or placebo for up to 36 months (21). Of the original cohort, 50 subjects completed 12 months, 41 completed 24 months, and 28 completed 36 months. At study completion, linear growth was comparable between both groups; however, after 2 years, there was a significantly slower mean increase in BA advancement from baseline in the anastrozole vs the placebo group (+1.8 vs +2.78 years, p<0.0001) and after 3 years (+2.58 vs +4.18 years, p<0.0001). This difference resulted in a net increase in mean predicted adult height of 4.5 cm in the anastrozole group at 24 months and 6.8 cm at 36 months compared with a 1-cm gain at both time points in placebo group. During the course of the study, all boys on AIs had a normal tempo of pubertal virilization and, as expected, E2 and E1 concentrations increased less in the anastrozole vs the placebo group.

## SAFETY OF AROMATASE INHIBITORS

In the Finnish trials (17, 22, 23), over the first 2 years of study, no differences in the rate of occurrence of adverse events between treatment groups were recorded involving multiple safety parameters, including lipids, adiponectin, GH surrogates, insulin sensitivity by homeostatic model assessment (HOMA), skinfold thickness, and BMD, with the exception of reduced levels of HDL cholesterol and fat mass in the letrozole-treated group. Of note, however, mean T levels were 455 ng/dL in T and placebo, and 1415 ng/dL in T and letrozole at 12 months. In the longer term study of this cohort, Hero et al showed an increase in bone mineral apparent density (employing a volumetric correction) by DEXA scan in letrozole-treated boys with ISS after 2 years, but not in the placebo-treated group (24). Furthermore, in the study of Karmazin et al, ˜25% of letrozole-treated subjects showed asymptomatic biochemical evidence of adrenal suppression (19). 

Additional theoretical adverse effects of induced estrogen depletion and/or androgen excess (for which data exist) must also be considered (16). With regard to possible growth suppression, Hero et al has reported lower serum IGF-I levels in letrozole-treated teenagers (25). Possible effects of this altered hormonal milieu on cognition are based on three pieces of information. First, verbal memory and estrogen levels have been noted to change in parallel during the menstrual cycle. Second, in a cross-sectional study of women with breast cancer, those treated with anastrazole performed poorly on tests of verbal memory function compared to controls. Third, healthy older men treated with testosterone improved spatial and verbal memory, findings negated with addition of anastrazole.

Perhaps the area that needs the most consideration is the possible long-term effect on spermatogenesis and fertility. Such concerns are based on the fact that estrogen receptor-a knock-out male mice show progressive impairment of fertility due to loss of the fluid resorptive function in epithelial cells of efferent ductules, resulting in their swelling and swelling of the rete testes (26). In addition, male aromatase knock-out mice have impaired spermatogenesis in older age. Furthermore, treatment of male monkeys with letrozole reduces sperm count and quality. In contradistinction, infertile men with low T:E2 ratios had improved sperm count and quality after treatment with anastrazole. Specifically with regard to potential reproductive effects of AI use in adolescent males, Mauras et al examined sperm concentration, motility, and morphology in 11 adolescent males (mean age 18.1 years) with GH deficiency treated with anastrazole 29 months prior on average and found no differences compared to controls, although sperm counts were somewhat low in both groups, raising into question validity of their laboratory methodology (27).

With regard to possible AI effects on bone turnover, letrozole-treated boys showed an initial increase followed by a slow declined in the bone resorption marker, urine aminoterminal telopeptide of type I collagen, while serum concentrations of the bone formation markers, s-PINP and alkaline phosphatase remained unchanged (25). In contrast, all markers of bone turnover increased significantly in the placebo-treated boys. In addition, increased concentrations of androgens have been shown to inhibit osteoclast differentiation and bone resorption in humans, as well as in cultured human osteoclasts.

A new concern that has recently arisen is the possible effect of AIs on vertebral morphology. Hero et al recently reported the detection of asymptomatic or mildly symptomatic vertebral wedge deformities in boys with ISS previously treated with letrozole vs placebo (25). Using Dual X-ray Absorptiometry-Instant Vertebral Assessment (DXA-IVA), wedge deformities were classified as mild (Grade 2a) when anterior vertebral height reduction is >20%, but <50%, and as severe (Grade 2b), when reduction is >50%. In mild and severe compression deformities, anterior, middle, and posterior vertebral heights are decreased by 20-30% (Grade 3a) and by >30% (Grade 3b), respectively. Of note, 6/13 (46%) letrozole-treated boys and 4/11 (36%) placebo-treated boys had 14 and 9 involved vetrebrae, respectively, of which most were Grades 2a and 3a in both groups. Although there was no statistical difference between the occurrence rate in the two groups, baseline studies were not performed. Thus, in ISS, some boys may have a defect of bone metabolism that impairs both bone growth and strength and results in anterior vertebral wedge deformity. Further studies on the impact of aromatase inhibition on bone architecture and vertebral morphology are clearly needed.

Theoretical effects of estrogen depletion and/or androgen excess by AIs, for which meaningful data do not exist, might include a possible effect on sexual orientation. Finally, the possible use of AIs in girls has not been studied, although induced hyperandrogenemia in females would be likely to severely limit their use.

## SUMMARY AND CONCLUSIONS

As of the end of 2008, results (with none to adult height) from only ˜110 children worldwide with short stature treated with a variety of third-generation AIs (and usually other growth-promoting therapy) have been reported and only three randomized, double-blinded, placebo-controlled trials have been performed. Five of the original seven reports derive from the same two research groups and two of those involve same initial patients. There has been only one small prospective randomized trial of AIs as sole therapy in adolescent males with ISS, but none in boys with CDGP. Only nine patients have been followed to near-final height and there are no available data regarding adult heights from any of the controlled trials. Moreover, one of the studies involving patients with GH deficiency found no change in mean predicted adult height among patients who were treated with AIs. 

It appears that these formulations are well-tolerated and, over relatively short time periods (12-18 months), no “significant” side effects have been reported. While (theoretical) concerns remain regarding BMD accrual, bone morphology, HDL concentrations, spermatogenesis, cognitive function, and the metabolic effects of decreased circulating estradiol, AIs have been promising in delaying BA advancement and, in most, but not all studies, in improving predicted adult height.

If proven efficacious and safe, AIs would be a useful new pharmacological intervention for boys with rapid BA advancement, various forms of precocious puberty, and/or CDGP. However, due to the dearth of scientific data currently available on the long-term efficacy and safety of AI treatment in children and adolescents, the use of such therapy outside of research settings should be discouraged. Thus, well-designed, long-term studies of efficacy and safety of AIs are required and should be supported by the pharmaceutical industry and/or other granting agencies.
